# Inhibitory Mechanisms of *trans*-2-Hexenal on the Growth of *Geotrichum citri*-*aurantii*

**DOI:** 10.3390/jof9090930

**Published:** 2023-09-15

**Authors:** Qiuli Ouyang, Shiwei Shi, Yangmei Liu, Yanqin Yang, Yonghua Zhang, Xingxing Yuan, Nengguo Tao, Lu Li

**Affiliations:** School of Chemical Engineering, Xiangtan University, Xiangtan 411105, Chinaluli9003@163.com (L.L.)

**Keywords:** *G. citri*-*aurantii*, *trans*-2-hexeanl, antifungal mechanism, cell wall, cell membrane

## Abstract

*Geotrichum citri*-*aurantii* (*G. citri*-*aurantii*) is one of the most important postharvest pathogens leading to a postharvest loss of citrus by causing sour rot. In this study, the antifungal activity of *trans*-2-hexenal, a natural component of essential oil, against *G. citri*-*aurantii* was evaluated. *Trans*-2-hexenal treatment inhibited the mycelia growth of *G. citri*-*aurantii* with a minimum inhibitory concentration and minimum fungicidal concentration of *trans*-2-hexenal at 0.50 and 1.00 μL/mL, respectively. Moreover, *trans*-2-hexenal efficiently reduced the incidence of sour rot of Satsuma fruit inoculated with *G. citri*-*aurantii*. Ultrastructural observations and Fourier transform infrared (FT−IR) results showed that *trans*-2-hexenal treatment affected the cell wall and cell membrane instructions of *G. citri*-*aurantii*. The content of *β*-1,3-glucan was significantly decreased after *trans*-2-hexenal treatment, but the cell wall permeability was not changed. The decrease in lipid and ergosterol contents might be responsible for this antifungal activity. Several important genes, *FKS1*, *ERG1*, *ERG7*, and *ERG11*, showed decreasing expression levels after *trans*-2-hexenal treatment. Molecule-docking results also indicated that *trans*-2-hexenal could join with the protein of FKS1, ERG1, ERG7, and ERG11 to impact enzyme activities. These results demonstrated that *trans*-2-hexenal is a promising fungicide for controlling sour rot of harvested citrus fruit by damaging the membrane integrity of *G. citri*-*aurantii.*

## 1. Introduction

*G. citri-aurantii* is a necrotrophic fungal pathogen that infects citrus fruit, and it has the characteristics of strong infectivity, fast infectivity, and being difficult to control [1,2]. Synthetic fungicides are exclusively used to control this disease but cause serious hazardous effects on the fruit rind, the environment, and human health [3,4]. Thus, it is necessary to focus on developing alternatives to synthetic fungicides for handling and maintaining the quality of citrus fruit [5,6,7].

Previous studies have shown that plant essential oils and their antimicrobial components have significant inhibitory effects on many postharvest pathogenic fungi of citrus, such as *G. citri*-*aurantii*, *Penicillium digitatum*, and *P. italicum*, and that they have the advantages of safety, high efficiency, and low residue, which means they have the potential to control postharvest diseases in citrus [8,9]. *Trans*-2-hexenal is a volatile component of plant essential oil, which naturally exists in citronella oil, camphor oil, apples, and grapes. It is an important signal molecule for plants to respond to and defend against their external environment. Some studies have shown that *trans*-2-hexenal has good inhibitory effects against *Colletotrichum acutatum*, *Alternaria alternata*, *P. cyclopium*, *P. expansum,* and *Botrytis cinerea* [10,11,12,13]. In addition, *trans*-2-hexenal was also successfully applied in order to control postharvest diseases such as gray mold in tomato fruits, green mold in citrus fruits, and black rot in ‘Zaosu’ pears [9,14,15].

As mentioned above, several studies have shown that *trans*-2-hexenal is a potential biological alternative to other preservative methods of controlling postharvest diseases, but there are few studies related to the investigation of its exact mechanism of antifungal action. Studies have shown that *trans*-2-hexenal interferes with the cell wall structure of fungi. For example, Arroyo et al. [10] pointed out that treatment with *trans*-2-hexenal can cause obvious cracks in the cell wall, disorder cell components to a high degree, and cause the organelle morphology to disappear and the cells to crack. Research by Zhang et al. [13] found that the membrane permeability of *P. cyclopium* increased with an increase of *trans*-2-hexenal concentration, resulting in the release of cell components and the leakage of potassium ions. At the same time, the integrity of the cell membrane of *P. cyclopium* was destroyed due to the decrease in the lipid content. Ma et al. [16] showed that 1.0 μL/mL *trans*-2-hexenal inhibited spore germination by disrupting the mitochondrial energy metabolism of *Aspergillus flavus*; the *trans*-2-hexenal treatment also decreased the acetyl-CoA and ATP contents, and the mitochondrial dehydrogenases activity increased by 65.7 ± 3.7%, 53.9 ± 4.0%, and 23.8 ± 2.2%, respectively. However, the antifungal activity of *trans*-2-hexenal against *G. citri*-*aurantii* has not been determined, and the antifungal mechanism of *trans*-2-hexenal against *G. citri*-*aurantii* has not been studied.

Thus, this study aimed to (1) study the antifungal properties of *trans*-2-hexenal against *G. citri*-*aurantii* in vitro and in vivo, (2) investigate the effect of the cell wall and cell membrane of *G. citri*-*aurantii* in the presence of *trans*-2-hexenal, and (3) further explore the possible mechanism through RT-qPCR and molecular docking.

## 2. Materials and Methods

### 2.1. Pathogen

*G. citri*-*aurantii* was provided by the Department of Biotechnology and Food Engineering, Xiangtan University, Xiangtan, China. The fungus was purified and preserved at 28 ± 2 °C on potato dextrose agar (PDA). A spore suspension (5 × 10^6^ spores/mL) in potato dextrose broth (PDB) was prepared using a hemocytometer.

### 2.2. Fruit

Satsuma mandarin fruits (*Citrus unshiu* Marc. cv. Miyagawa Wase) were harvested on 18 October 2018 from an orchard in Xiangtan, Hunan, China. Healthy fruits of uniform size and without scars were selected for the experiments.

### 2.3. Chemicals

*Trans*-2-hexenal (98%) was obtained from Aladdin (Shanghai, China). All the chemicals were analytical grade.

### 2.4. Antifungal Activity of trans-2-Hexenal against G. citri-aurantii

The inhibition of *trans*-2-hexenal on the growth of *G. citri*-*aurantii* mycelia was tested in vitro through the agar dilution method [17]. Briefly, *trans*-2-hexenal solutions were prepared by dissolving the requisite amount in Tween-80 (0.5%, Aladdin, Shanghai, China) and adding it to PDA (20 mL) to achieve the desired concentrations (0, 0.25, 0.50, 1.0, 2.0, and 4.0 μL/mL). A 6 mm diameter mycelial disk of inoculate was cut from the actively growing culture of the PDA plates. Then, they were placed at the center of each new Petri plate (90 mm in diameter). The culture plates were then incubated at 28 ± 2 °C for 2 d. Each treatment was performed in triplicate. The percentage of inhibition of mycelial growth (MGI) was calculated according to the following formula:

MGI (%) = [(dc − dt)/(dc − 6)] × 100%

where dc (cm) is the average diameter of the control and dt (cm) is the average diameter of the treatment. The lowest concentration that completely inhibited the growth of *G. citri*-*aurantii* after 2 d of incubation was considered to be the minimum inhibitory concentration (MIC). The minimum fungicidal concentration (MFC) was regarded as the lowest concentration that prevented the growth of the pathogen after 4 d of incubation at 28 ± 2 °C, indicating that more than 99.5% of the original inocula were killed.

### 2.5. In Vivo Experiments of trans-2-Hexenal against G. citri-aurantii

The effect of the *trans*-2-hexenal on the incidence of sour rot was determined as described previously by Dou et al. [18]. All fresh citrus fruit were surface-sterilized by immersing in 2% sodium hypochlorite solution (*v*/*v*) for 2 min, then washed with distilled water, wounded (depth of 3 mm and width of 3 mm) with a sterile needle, inoculated with 20 μL of *G. citri*-*aurantii* spore suspension (10^5^ spores mL^−1^), and left to air-dry. After being inoculated with *G. citri*-*aurantii*, the fruit were soaked in wax amended with *trans*-2-hexenal at 1× MFC and 10× MFC. The fruit with wax and inoculated with the pathogen inoculation was used as a control. The inoculated fruit was kept in sealed incubators at 25 ± 2 °C to ensure a high relative humidity (80–85% relative humidity). Each treatment was performed in triplicate, and each replicate contained 20 Satsuma fruits. The incidence rate of disease (measured by counting the number of green-mold-infected wounds) was calculated as follows:

$$\mathrm{Disease}\mathrm{incidence}=\frac{Number\;of\;rotten\;wounds}{Total\;number\;of\;wounds}\times 100\%$$

### 2.6. Scanning Electron Microscopy (SEM) of trans-2-Hexenal against G. citri-aurantii

The mycelia treated with *trans*-2-hexenal for 30 min, as described above, were directly examined using a JEOL JSM-6360LV SEM instrument (JEOL, Tokyo, Japan). The hyphae grown on PDA without *trans*-2-hexenal were used as a control. The procedures for the SEM observation were described in our previous study [19].

### 2.7. Transmission Electron Microscopy (TEM) of trans-2-Hexenal against G. citri-aurantii

The mycelia treated with *trans*-2-hexenal for 30 min, as described above, were directly examined using a transmission electron microscope (JEM-1230; JEOL Ltd., Tokyo, Japan) operated at an accelerating voltage of 80 kV. The hyphae grown on PDA without *trans*-2-hexenal were used as a control. The procedures for the TEM observation were described in our previous study [19].

### 2.8. Fourier Transform Infrared (FT−IR) Spectroscopy of trans-2-Hexenal against G. citri-aurantii

The effect of *trans*-2-hexenal on the mycelia composition of *G. citri*-*aurantii* was analyzed using Fourier transform infrared spectroscopy (FT−IR) (Thermo Fisher Scientific, Waltham, MA, USA) [20]. The mycelia treated with 1/2 MIC *trans*-2-hexenal for 30 min were collected, frozen with liquid nitrogen, and then vacuum freeze-dried. Subsequently, the mycelia were ground (100 mesh) to obtain uniform dried powder. The samples were prepared using the potassium bromide-disk technique for the FT−IR detection. The scanning range was 4000–400 cm^−1^ with the resolution of 4 cm^−1^ and 128 separate scans. The infrared spectrum was analyzed using Unscrambler X (Version 10.4).

### 2.9. Effect of trans-2-Hexenal on the Cell Wall of G. citri-aurantii

The effects of *trans*-2-hexenal on the cell wall integrity of *G. citri*-*aurantii* were analyzed using calcofluor white (Sigma, St. Louis, MO, USA) staining coupled with fluorescence microscopy. The mycelia treated with 1/2 MIC *trans*-2-hexenal for 0, 30, 60, and 120 min were centrifuged at 4000× *g* for 10 min. The collected mycelia were stained with 10 μL of calcofluor white stain after the addition of 10 μL KOH (10%) following the manufacturer’s instructions. The samples were observed with a fluorescence microscope (Nikon ECLIPSE TS100, Tokyo, Metropolis, Japan). The fungal culture in PDB without *trans*-2-hexenal was used as a control.

### 2.10. Effect of trans-2-Hexenal on the Cell Wall of G. citri-aurantii

#### 2.10.1. Effect of *trans*-2-Hexenal on the Cell Wall Integrity of *G. citri-aurantii*

The effects of *trans*-2-hexenal on the cell wall integrity of *G. citri*-*aurantii* were analyzed using calcofluor white (Sigma, St. Louis, MO, USA) staining coupled with fluorescence microscopy. The mycelia treated with 1/2 MIC *trans*-2-hexenal for 0, 30, 60, and 120 min were centrifuged at 4000× *g* for 10 min. The collected mycelia were stained with 10 μL of calcofluor white stain after the addition of 10 μL KOH (10%, Aladdin, Shanghai, China) following the manufacturer’s instructions. The samples were observed with a fluorescence microscope (Nikon ECLIPSE TS100, Tokyo Metropolis, Japan). The fungal culture in PDB without *trans*-2-hexenal was used as a control.

#### 2.10.2. Effect of *trans*-2-Hexenal on the Chitin Content of *G. citri-aurantii*

The chitin contents of the *G. citri*-*aurantii* treated with the 1/2 MIC *trans*-2-hexenal treatments in PDB were determined using the method of Francois [21]. A total of 0.5 g of dried mycelia was soaked in 4 mL of concentrated HCl (Soleibao, Beijing, China) at 25 °C for 24 h, then diluted with distilled water until the HCl reached a concentration of 8.5 mol/L, and the solution was further digested in a boiling water bath. After cooling, it was adjusted to neutral with 1 mol/L of NaOH (Aladdin, Shanghai, China) solution, the volume was made constant to 100 mL, then filtered with filter paper, and the supernatant was shaken to obtain the sample to be tested. Then, 200 μL supernatant was mixed with 400 μL acetylacetone reagent and placed in a 90 °C water bath for 1 h. After cooling to room temperature, 4 mL absolute ethyl alcohol and 400 μL 4-dimethylaminobenzaldehyde were added to the supernatant, the volume was fixed to 5 mL with absolute ethyl alcohol, and it was left to stand at room temperature for 1 h. The absorbance of the solution was measured at a wavelength of 530 nm, and glucosamine hydrochloride was used as the standard curve.

#### 2.10.3. Effect of *trans*-2-Hexenal on the *β*-1,3-Glucan Content of *G. citri-aurantii*

The method of Fortwendel et al. [22] was used for the determination of *β*-1,3-glucan. The mycelia were washed with 0.1 mol/L NaOH solution and then freeze-dried and ground into powder. A certain amount of powder was added to 1 mol/L NaOH solution, ultrasonicated for 30 s, placed in a water bath at 52 °C for 30 min, and centrifuged for 5 min after cooling. A total of 50 μL of the supernatant was taken, 185 μL of aniline blue solution was added, and it was placed in a water bath at 52 °C for 30 min and left to stand for 30 min. The fluorescence value (excitation wavelength: 405 nm; emission wavelength: 460 nm) was measured using a fluorescence spectrophotometer (Lengguang Technology Co., Ltd., Shanghai, China).

#### 2.10.4. Effect of *trans*-2-Hexenal on the Extracellular Alkaline Phosphatase (AKP) Activities of *G. citri-aurantii*

The extracellular AKP activities of the *G. citri*-*aurantii* mycelia that received different *trans*-2-hexenal treatments in PDB, as described above, were assayed with a UV-2450 UV/V spectrophotometer (Shimadzu (China) Co., Ltd., Shanghai, China) using a commercially available kit, following the instructions. The fungal culture in PDB without *trans*-2-hexenal was used as a control. Each experiment was repeated three times. The enzyme activity is expressed as U/g prot.

### 2.11. Effect of trans-2-Hexenal on the Cell Membrane of G. citri-aurantii

#### 2.11.1. Effect of *trans*-2-Hexenal on the Cell Membrane Integrity of *G. citri-aurantii*

The cell membrane integrity of the *G. citri*-*aurantii* that received different *trans*-2-hexenal treatments in PDB, as described above, was analyzed using propidium iodide (PI) staining coupled with an ECLIPSE TS100 microscope (Nikon, Tokyo Metropolis, Japan) and F97 PRO fluorescence spectrophotometer (Lengguang Technology, Shanghai, China) [23].

#### 2.11.2. Effect of *trans*-2-Hexenal on the Total Lipid Content of *G. citri-aurantii*

The total lipid content of *G. citri*-*aurantii* cells with *trans*-2-hexenal at various concentrations (0 and 1/2 MIC) for 0, 30, 60, and 120 min was determined using the phosphovanillin method [19]. The fungal culture in PDB without *trans*-2-hexenal was used as a control.

#### 2.11.3. Effect of *trans*-2-Hexenal on the Ergosterol Contents of *G. citri-aurantii*

The ergosterol contents of *G. citri*-*aurantii* cells that received different *trans*-2-hexenal treatments in PDB, as described above, were determined using the HPLC method [23]. The fungal culture in PDA without *trans*-2-hexenal was used as a control.

### 2.12. Real-Time Fluorescence Quantitative PCR (RT-qPCR) Analysis

The effects of *trans*-2-hexenal on the transcriptional profiles of *FKS1* (the key gene that synthesizes *β*-1,3-glucan synthase) and genes related to ergosterol synthesis (*ERG1*, *ERG7*, and *ERG11*) in *G. citri*-*aurantii* were evaluated, and the sequences were obtained from a previous RNA-Seq of *G. citri*-*aurantii*. RNA was extracted from *G. citri*-*aurantii* cells exposed to *trans*-2-hexenal at concentrations of 0 and 1/2 MIC for 0, 30, 60, and 120 min using Trizol reagent (Invitrogen, Carlsbad, CA, USA) following the manufacturer’s instructions. Two micrograms of DNA-free RNA were used for the reverse transcription using M-MLV (Promega, Madison, WI, USA) with oligo dT18. The RT-qPCR was performed on a BIO-RAD CFX Connect Thermal Cycler using FastStart Universal SYBR Green Master (Roche, Basel, Switzerland). All primer pairs for the expression assays are listed in Table 1. The RT-qPCR was programmed as follows: initial denaturation at 95 °C for 10 min, followed by 40 cycles of denaturation at 95 °C for 15 s and a combined annealing and extension step at 60 °C for 1 min. The 2^−ΔΔCT^ method was used to quantify the value of every sample using the actin gene as an internal reference [24].

### 2.13. Molecular Docking

Selecting FKS1, ERG1, ERG7, and ERG11 as the receptors and using *Alphafold2* (https://colab.research.google.com/github/sokrypton/ColabFold/blob/main/AlphaFold2ipynb; accessed on 8 July 2022) for the homology modeling, the structure of the models were optimized using *ModRefiner* (https://zhanggroup.org/ModRefiner/; accessed on 8 July 2022), and the obtained models were evaluated with *SAVES v6.0* (https://saves.mbi.ucla.edu/; accessed on 20 July 2022). In this way, the three-dimensional protein structure models of ERG1, ERG7, ERG11, and FKS1 were obtained. The structure of the ligand small molecule *trans*-2-hexenal (CID: 5281168; MF: C_6_H_10_O) was obtained from the chemical structure database of the *PubChem* (https://pubchem.ncbi.nlm.nih.gov/; accessed on 20 July 2022) website. Using *PyMol* (Version 2.5.2) to process the receptor protein, the water molecules and metal ions were deleted. The *Openbabel* module in *PyRx* (Version 0.8) was used to minimize the ability of the ligands. The *Autodock Vina* module in *PyRx* was used for the molecular docking. The optimal docking model was selected according to the binding energy. *PyMol* and *Ligplot+* (Version 2.2.8) were used to connect the three-dimensional and two-dimensional visual analysis of the model.

### 2.14. Statistical Analyses

All data are expressed as the mean ± SD (standard deviation), and they were measured using three independent replicates and analyzed using one-way analysis of variance (ANOVA) followed by Duncan’s test. A value of *p* < 0.05 was considered statistically significant using SPSS statistical software package release 16.0 (SPSS Inc., Chicago, IL, USA).

## 3. Results

### 3.1. Antifungal Activity of trans-2-Hexenal against G. citri-aurantii

Table 2 shows the effect of *trans*-2-hexenal on the mycelial growth of *G. citri*-*aurantii* in vitro. The results show that the mycelial growth considerably decreased with an increasing *trans*-2-hexenal concentration and incubation time. Mycelia growth was inhibited to different degrees at 0.25 μL/mL to 0.50 μL/mL of *trans*-2-hexenal. At a concentration of 0.50 μL/mL, the growth of *G. citri*-*aurantii* was completely inhibited after 2 d of incubation. As the duration of the culture was prolonged to 4 d, 72.9 ± 3.9% and 100.0 ± 0.0% of the mycelial growth was inhibited by 0.50 and 1.00 μL/mL of *trans*-2-hexenal, respectively. Thus, the MIC and MFC of *trans*-2-hexenal were 0.50 and 1.00 μL/mL, respectively.

### 3.2. In Vivo Experiments of trans-2-Hexenal against G. citri-aurantii

**Figure 1:**
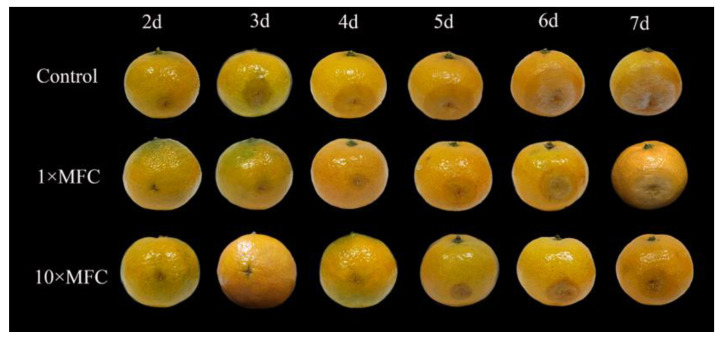
Disease progression in inoculated citrus fruit treated with *trans*-2-hexenal (0×, 1× and 10× MFC) during storage at 25 ± 2 °C and 80–85% RH. The data presented are the means of pooled data. Error bars indicate the SDs of the means (*n* = 20).

*trans*-2-Hexenal (1× and 10× MFC) effectively reduced the decay of citrus fruit inoculated with *G. citri*-*aurantii* (Table 3), and the disease progression in the inoculated citrus fruit treated with *trans*-2-hexenal is presented in Figure 1. The control group began to decay within 2 d with 11 ± 4% decay, while the citrus fruit remained healthy after treatment with *trans*-2-hexenal. The citrus fruit in the 1× and 10× MFC *trans*-2-hexenal groups began to rot after 3 d and 5 d of treatment, respectively. After 7 d of storage, the incidence of fruit in the fruit wax control group reached 100%, while that in the 1× and 10× MFC treatment groups were only 85 ± 4% and 33 ± 12%, respectively.

### 3.3. Scanning Electron Microscopy (SEM) of trans-2-Hexenal against G. citri-aurantii

**Figure 2:**
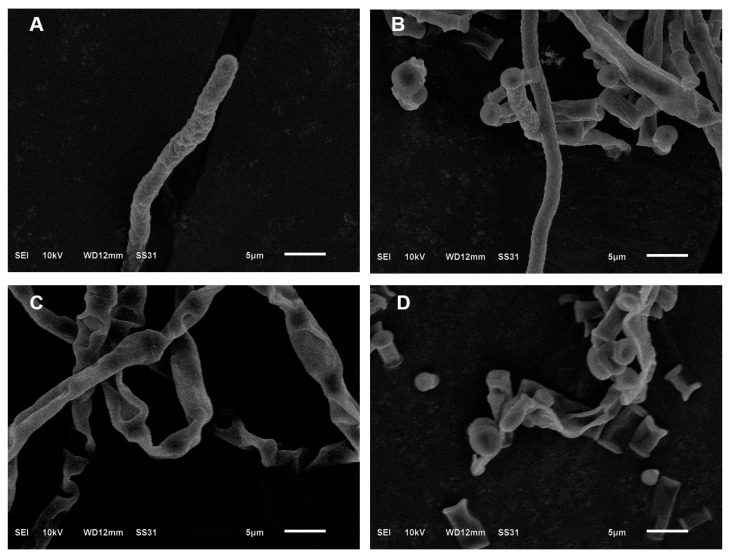
SEM images of (**A**,**B**) untreated control culture of *G. citri*-*aurantii*; (**C**,**D**) culture after incubation with the *trans*-2-hexenal.

The effects of *trans*-2-hexenal on the surface morphology of *G. citri*-*aurantii* are shown in Figure 2. The mycelia in the control group were regular in shape, uniform in thickness, smooth on the surface, healthy, and full (Figure 2A,B). However, after the 1/2 MIC *trans*-2-hexenal treatment, the surface of the mycelia became wrinkled and severely twisted, shrank, and collapsed (Figure 2C,D). The results show that *trans*-2-hexenal can change the morphology of mycelia of *G. citri*-*aurantii*.

### 3.4. Transmission Electron Microscopy (TEM) of trans-2-Hexenal against G. citri-aurantii

**Figure 3:**
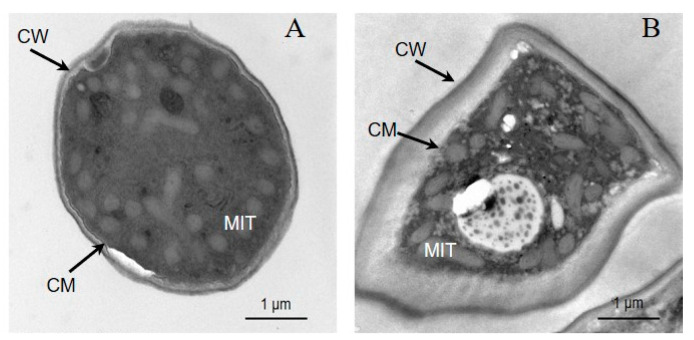
TEM images of (**A**) untreated control culture of *G. citri*-*aurantii*; (**B**) culture after incubation with the *trans*-2-hexenal. CW: cell wall; CM: cell membrane; MIT: mitochondrion.

The effects of *trans*-2-hexenal on the internal morphology of *G. citri*-*aurantii* are shown in Figure 3. In the control group, the cells were composed of uniform cell walls, cell membranes, and cytoplasm, with uniform organelles and a complete structure (Figure 3A). After the 1/2 MIC *trans*-2-hexenal treatment, the internal morphology and ultrastructure of the *G. citri*-*aurantii* cells were destroyed, the cell walls became thicker, the cell membranes were irregularly twisted and, in some areas, the organelle structures were disordered, and the mitochondria were enlarged with irregular distribution (Figure 3B).

### 3.5. FT−IR of trans-2-Hexenal against G. citri-aurantii

**Figure 4:**
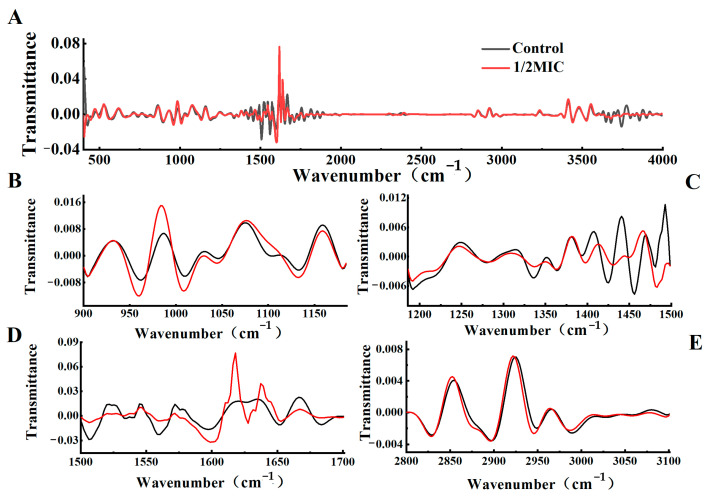
FT−IR spectra analysis of *G. citri*-*aurantii* under *trans*-2-hexenal treatment. (**A**) FT−IR spectra of the second derivative (400–4000 cm^−1^); (**B**) the second derivative of FT−IR spectra of the nucleic acid and polysaccharide region (900−1200 cm^−1^); (**C**) the second derivative of FT−IR spectra of the mixed region of fatty acid, protein, and polysaccharide (1200−1500 cm^−1^); (**D**) the second derivative of FT−IR spectra of the mixed region (1500−1700 cm^−1^); (**E**) the second derivative of FT−IR spectra of the lipid region (2800−3100 cm^−1^).

In the region of >3000 cm^−1^, after the *trans*-2-hexenal treatment, the O–H expansion (3775 cm^−1^) of alcohols in the carbohydrates shifted, suggesting that the *trans*-2-hexenal treatment may change the components of the cell walls and cell membranes of *G. citri*-*aurantii* (Figure 4). The N-H stretching (3415 cm^−1^) of the amino groups and amide groups also shifted, which may be related to the Michael addition reaction between the *α*,*β*-unsaturated carbonyl groups in *trans*-2-hexenal and the amino groups in protein, resulting in the degradation of the cell wall proteins. In the 2800–3100 cm^−1^ region, *trans*-2-hexenal caused a significant shift of the C-H asymmetric stretching (2928 cm^−1^) in the methyl and acyl chains, which might be related to the changes in the fatty acids caused by the condensation of the active methylene-containing compounds with aldehydes. The amides in the polypeptides and the protein (1584 cm^−1^ and 1637 cm^−1^) in the 1500–1700 cm^−1^ region all shifted, which might be related to the peroxidation and degradation of protein. In the range of 900–1500 cm^−1^, the peak positions at 1400 cm^−1^ and 1077 cm^−1^, corresponding to fatty acids, proteins, polysaccharides, and nucleic acid, shifted after the treatment with *trans*-2-hexenal, suggesting that the cell wall’s polysaccharides and nucleic acid were affected by *trans*-2-hexenal.

### 3.6. Effect of trans-2-Hexenal on the Cell Wall of G. citri-aurantii

**Figure 5:**
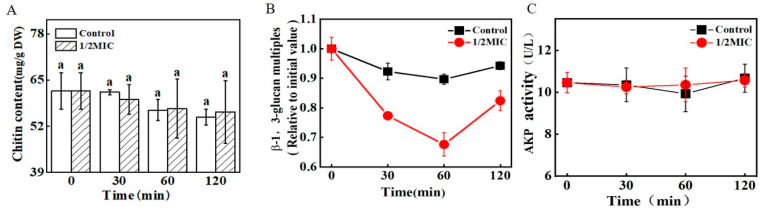
The effects of *trans*-2-hexenal on the cell walls of *G. citri*-*aurantii*. (**A**) The chitin content of *G. citri*-*aurantii*; (**B**) the *β*-1,3-glucan content of *G. citri*-*aurantii*; (**C**) the extracellular AKP activity of *G. citri*-*aurantii*. The data presented are the means of pooled data. Error bars indicate the SDs of the means (*n* = 3). “a” indicated that there was no difference between different treatment groups (*p* < 0.05).

Figure 5A shows the effect of *trans*-2-hexenal on the chitin content of *G. citri*-*aurantii*. There was no significant difference in the chitin content between the *trans*-2-hexenal-treated group and the control group, suggesting that *trans*-2-hexenal had no effect on the chitin content of *G. citri*-*aurantii*.

The content of *β*-1,3-glucan in the *trans*-2-hexenal-treated group was significantly lower than that in the control group (Figure 5B). At 30 min, the *trans*-2-hexenal-treated group decreased by 17.31% compared with the control group (*p <* 0.05), which indicates that the content of *β*-1,3-glucan significantly reduced after *trans*-2-hexenal treatment.

The activity of extracellular AKP after *trans*-2-hexenal treatment had no significant difference with the control group (*p <* 0.05, Figure 5C), which further indicates that *trans*-2-hexenal did not damage the cell wall integrity of *G. citri*-*aurantii*.

### 3.7. Effect of trans-2-Hexenal on the Cell Membrane of G. citri-aurantii

**Figure 6:**
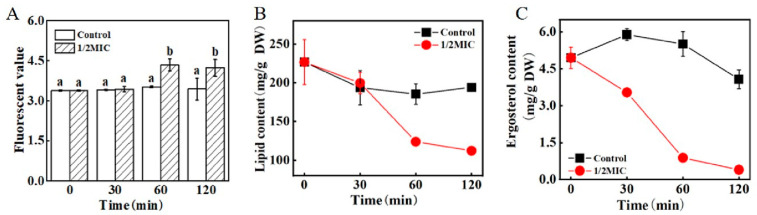
The effects of *trans*-2-hexenal on the cell membranes of *G. citri*-*aurantii*. (**A**) The plasma membrane integrity of *G. citri*-*aurantii* mycelia and mycelia fluorescence times; (**B**) the total lipids contents and (**C**) ergosterol contents of *G. citri*-*aurantii* mycelia. The data presented are the means of pooled data. Error bars indicate the SDs of the means (*n* = 3). “a,b” indicates the difference among different treatment groups (*p* < 0.05).

According to the results of the PI staining (Figure 6A), the fluorescence value of the mycelia treatment group after the 1/2 MIC *trans*-2-hexenal treatment for 60 min was 1.24 times that of the control group, which was significantly higher than that of the control group (*p* < 0.05), indicating that the integrity of the cell membrane might begin to be destroyed after 60 min.

The effect of *trans*-2-hexenal on the total lipid content of *G. citri*-*aurantii* is shown in Figure 6B. After the 1/2 MIC *trans*-2-hexenal treatment for 60 min, the total lipid content was 123.8 ± 1.6 mg/g DW, which was significantly lower than that of the control group (185.4 ± 13.3 mg/g DW) (*p* < 0.05), suggesting that the integrity of the cell membrane of *G. citri*-*aurantii* was destroyed, and the cells were damaged after the 1/2 MIC *trans*-2-hexenal treatment for 60 min.

The ergosterol content (3.54 ± 0.07 mg/g DW) of *G. citri*-*aurantii* was significantly lower than that of the control group (5.89 ± 0.24 mg/g DW) (*p* < 0.05) after 30 min of treatment with *trans*-2-hexenal, suggesting that *trans*-2-hexenal could affect the synthesis of ergosterol (Figure 6C).

### 3.8. RT-qPCR

**Figure 7:**
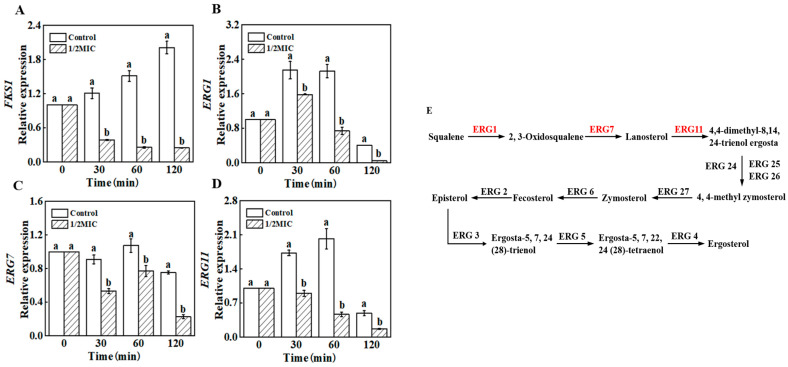
Changes in the expression of the *β*-1,3-glucan and ergosterol biosynthesis genes of *G. citri*-*aurantii* by control and 1/2 MIC of *trans*-2-hexenal for 0, 30, 60, and 120 min ((**A**) *FKS1*; (**B**) *ERG1*; (**C**) *ERG7*; (**D**) *ERG11*). (**E**) Ergosterol biosynthesis pathway. The data presented are the means of pooled data. Error bars indicate the SDs of the means (*n* = 3). “a,b” indicates the difference among different treatment groups (*p* < 0.05).

In order to determine the effect of *trans*-2-hexenal on *β*-1,3-glucan and ergosterol, the key biosynthesis genes related to *β*-1,3-glucan biosynthesis and ergosterol biosynthesis were selected for RT-qPCR analysis. As revealed in Figure 7, the expression levels of *FKS1*, *ERG1*, *ERG7*, and *ERG11* were significantly lower than the control samples in the whole *trans*-2-hexenal treatment period.

### 3.9. Molecular Docking

**Figure 8:**
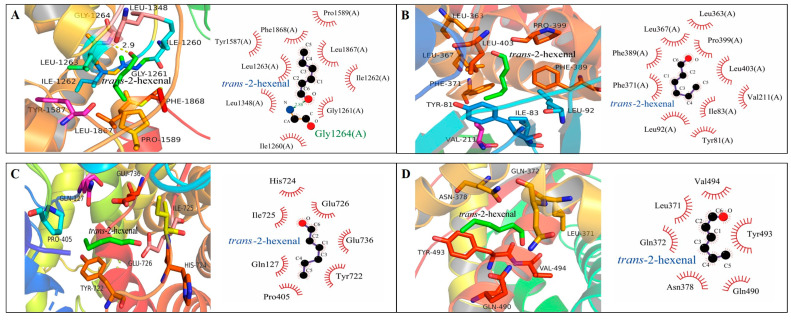
The 3D images of FKS1 (**A**), ERG1 (**B**), ERG7 (**C**), and ERG11 (**D**) proteins and the 2D interaction diagram of *trans*-2-hexenal with FKS1 (**A**), ERG1 (**B**), ERG7 (**C**), and ERG11 (**D**) proteins. The dark green rod-like structure in the diagram is *trans*-2-hexenal, and the other color rod-like structures are amino acid residues.

Using *Alphafold2*, FKS1, ERG1, ERG7, and ERG11 were homology modeled; then, *trans*-2-hexenal was docked against the FKS1, ERG1, ERG7, and ERG11 protein, respectively (Figure 8). The binding energies of *trans*-2-hexenal to the best conformations of FKS1, ERG1, ERG7, and ERG11 were −4.9 kcal/mol, −4.6 kcal/mol, −4.1 kcal/mol, and −3.9 kcal/mol, respectively. Because of the different binding conformations of the ligand molecules in the conjugates, the amino acid residues around the active sites of their interactions and the strength of their interactions were different. *trans*-2-Hexenal formed a hydrophobic interaction with Ile1260, Gly1261, Ile1262, Leu1263, Leu1348, Tyr1587, Pro1589, Phe1868, and Leu1867 in FKS1, while the aldehyde group (-COH) of *trans*-2-hexenal acted as an acceptor to form a hydrogen bond with a bond length of 2.88 Å with the side chain of Gly1264 in FKS1; the binding of *trans*-2-hexenal to FKS1 with that hydrophobic interaction and hydrogen bonding promote the binding of *trans*-2-hexenal to FKS1. trans-2-Hexenal formed hydrophobic interactions with amino acid residues Pro399, Leu363, Leu403, Tyr81, Leu367, Val211, Ile83, Leu92, Phe37, and Phe389 in ERG1; His724, Ile725, Glu726, Glu736, Gln127, Tyr722, and Pro405 in ERG7; and Leu371, Val494, Gln490, Tyr493, Asn378, and Gln372 in ERG11. Hydrophobic interactions provide the main driving force for the combination of *trans*-2-hexenal with ERG1, ERG7, and ERG11, which results in altered interactions among amino acid residues within the protein molecule and plays an important role in maintaining protein conformation.

## 4. Discussion

*G. citri*-*aurantii* is considered one of the most important pathogens in terms of the economic losses that hinder citrus worldwide [2,18,19]. In the present study, *trans*-2-hexenal showed pronounced antifungal efficacy against *G. citri*-*aurantii* in vitro and in vivo. These results are consistent with those of previous studies with regard to the antifungal activity of *trans*-2-hexenal [9,10,13,15], indicating that the application of *trans*-2-hexenal is a potent method for controlling *G. citri*-*aurantii*.

The fungal cell wall is a dynamic structure that protects cells from osmotic pressure and other environmental stresses. It is of great significance to the growth of fungal cells. The destruction of the cell wall’s structure easily leads to the cell’s dissolution and death. Plant essential oils, such as tea tree essential oil, *Hyssopus officinalis* essential oil, cinnamaldehyde, and thymol, can destroy the cell wall of fungi [10,25,26]. Cinnamaldehyde mainly inhibits the growth of *G. citri*-*aurantii* by inhibiting chitin synthesis, accelerating chitin hydrolysis, and destroying the integrity of the cell wall [19]. In this study, *trans*-2-hexenal treatment did not change the chitin content and cell wall integrity, but the *β*-1,3-glucan content of *G. citri*-*aurantii* decreased significantly. These results are similar to the results of Li et al. [27] in which o-vanillin mainly reduced the *β*-1,3-glucan content in the cell wall of *A. flavus*, but the content of chitin did not change. FKS family genes (*FKS1*, *FKS2*, and *FKS3*) are the key genes that synthesize *β*-1,3-glucan synthase, and *FKS1* is an essential gene, in most, that can regulate *β*-1,3-glucan synthase activity [28]. García et al. [29] found that the deletion of *FKS1* in yeast would lead to a decrease in the glucan content and slow the growth of the strain. The RT-qPCR results show that *trans*-2-hexenal decreased the expression of *FKS1*, which is consistent with the decrease in the *β*-1,3-glucan content, indicating that the *β*-1,3-glucan synthesis pathway was inhibited. Inhibitors of *β*-1,3-glucan synthase affect gene expression by targeting key groups of synthases, which, in turn, leads to a decrease in the content of *β*-1,3-glucan in the cell wall, resulting in cell rupture and death [30]. Douglas et al. [31] found that echinocandin binds to the catalytic subunit of *β*-1,3-glucan synthase to reduce the integrity of the fungal cell wall. Molecular docking showed that the hydrogen bond and hydrophobic interaction between *trans*-2-hexenal and FKS1 lead to a change in the FKS1 protein’s structure, rotatable bond dihedral angle, and amino acid residue side chain, which reduces its expression and interferes with the normal function of FKS1. *Trans*-2-hexenal inhibited the synthesis of the cell wall by combining different amino acid residues with the active site of FKS1 in the cell wall of *G. citri*-*aurantii*, which is consistent with the antibacterial mechanism of eugenol and citral against *P. roqueforti* and *A. niger* [32]. The decrease in *β*-1,3-glucan content in the cell wall may lead to an increase in fungal cell wall permeability, as evidenced by the increase in extracellular AKP activity of fungi [26]. However, despite the decrease in *β*-1,3-glucan content, the cell wall became thicker and the activity of extracellular AKP activity remained unchanged, suggesting that *trans*-2-hexenal did not destroy the cell wall permeability of *G. citri*-*aurantii* but induced its cell wall components to restructure, resulting in a decrease in the *β*-1,3-glucan content.

The integrity and fluidity of the cell membrane are crucial for the survival and growth of fungi, and they are key points in many kinds of drug treatment [33]. The fungal cell membrane is the protective barrier of fungal cells, and it is selective for substances to enter and leave the cells. Therefore, damage to the integrity of the cell membrane will cause the leakage of components, leading to cell apoptosis [34,35]. Lipids are an important component of the cell membrane, and they play important roles in cell membrane integrity and material transportation [36,37]. Ergosterol, as a unique component of fungal cell membranes, plays important roles in cell membrane fluidity and membrane protein function [38,39,40]. It was found that the synthesis of ergosterol in *A. flavus* cell membranes was significantly inhibited after being treated with *Artemisia annua* essential oil; thus, the fungal cell membranes were the main site [41]. After being treated with *trans*-2-hexenal, the lipid content of *G. citri*-*aurantii* decreased at 60 min. The ergosterol content in the treatment group decreased at 30 min, indicating that ergosterol was the primary target of *trans*-2-hexenal in inhibiting *G. citri*-*aurantii*. *ERG1*, *ERG7*, and *ERG11* are key genes in the sterol biosynthesis pathway, encoding squalene epoxidase, lanosterol synthase, and lanosterol-14-α-demethylase in the sterol biosynthesis pathway, respectively [19,42]. *ERG1*, *ERG7*, and *ERG11* in *G. citri*-*aurantii* were significantly downregulated after *trans*-2-hexenal treatment, which is consistent with the results of the ergosterol content, further indicating that the ergosterol synthesis pathway was attacked. Meanwhile, it is worth noting that the molecular docking analysis of the formed complexes shows that the formation of hydrophobic interactions may lead to a longer residence time of *trans*-2-hexenal in ERG1, ERG7, and ERG11, and the hydrophobic interactions may enhance the effect of *trans*-2-hexenal on the ergosterol synthesis pathway. This result is consistent with that of Song et al. [43], who found that *trans*-2-hexenal fumigation reduced the ergosterol content of *Botrytis cinerea* and influenced ergosterol biosynthetic gene expression levels.

## 5. Conclusions

In summary, *trans*-2-hexenal reduced the *β*-1,3-glucan content and ergosterol content of *G. citri*-*aurantii* by inhibiting the expressions of related genes. With the prolongation of the treatment time, the total lipid content decreased, and the cell membrane integrity was further destroyed.

## Figures and Tables

**Table 1:** Primer pair sequences designed for validation of differentially expressed genes in control and 1/2 MIC *trans*-2-hexenal treatment of *G. citri*-*aurantii* using RT-qPCR.

Gene ID	Genes	Primer Sequence (5′-3′)
*CL1729.Contig1_All*	*FKS1*-F	AGGTTGAAGGCAAGCGTACTCT
	*FKS1*-R	CAGGAAGTGGCTCAGGAATAGGT
*Unigene4828_All*	*ERG1*-F	AAGTCCTACACCTCCAAGGCTAC
	*ERG1*-R	GAATATCGGCGTCAGTGAGAACC
*Unigene3819_All*	*ERG7*-F	TAACGCATATCCAGGACGACCAA
	*ERG7*-R	CGCACAATCTCAATTCGCTCTTC
*Unigene3920_All*	*ERG11*-F	CGCCGTAAGGAAGGAAACATTGA
	*ERG11*-R	AAGACGAAGTAGCAGCCGAAGT
*CL313.Contig2*	*Actin1*-F	TTACGCCGGTTTCTCCCTCC
	*Actin1*-R	GACGATTTCACGCTCGGCAG

**Table 2:** Antifungal activity of *trans*-2-hexenal against *G. citri*-*aurantii.*

Concentration (µL/mL)	Inhibitory Rate (%)				
	1 d	2 d	3 d	4 d	5 d
0.25	100.0 ± 0.0 a	53.7 ± 5.2 b	24.8 ± 1.7 c	27.1 ± 3.0 c	23.2 ± 1.4 c
0.50	100.0 ± 0.0 a	100.0 ± 0.0 a	90.5 ± 1.7 b	72.9 ± 3.9 b	64.0 ± 8.7 b
1.00	100.0 ± 0.0 a	100.0 ± 0.0 a	100.0 ± 0.0 a	100.0 ± 0.0 a	100.0 ± 0.0 a
2.00	100.0 ± 0.0 a	100.0 ± 0.0 a	100.0 ± 0.0 a	100.0 ± 0.0 a	100.0 ± 0.0 a
4.00	100.0 ± 0.0 a	100.0 ± 0.0 a	100.0 ± 0.0 a	100.0 ± 0.0 a	100.0 ± 0.0 a

Note: Mean values ± SD (standard deviation) followed by different letters (a–c) represent significantly different scores in the same phase (*p* < 0.05).

**Table 3:** Effect of *trans*-2-hexenal on the incidence of Satsuma fruit inoculated with *G. citri*-*aurantii.*

Treatments	Incidence Rate (%)						
	1 d	2 d	3 d	4 d	5 d	6 d	7 d
Control	0 ± 0 a	11 ± 4 a	22 ± 4 a	49 ± 4 a	73 ± 0 a	89 ± 4 a	100 ± 0 a
1× MFC *trans*-2-hexenal	0 ± 0 a	0 ± 0 b	16 ± 4 b	22 ± 8 b	60 ± 7 b	80 ± 0 b	85 ± 4 b
10× MFC *trans*-2-hexenal	0 ± 0 a	0 ± 0 b	0 ± 0 c	9 ± 0 c	22 ± 8 c	29 ± 8 b	33 ± 12 c

Note: “a–c” indicates the difference among different treatment groups with the same storage time (*p* < 0.05).

## Data Availability

The datasets generated during and/or analyzed during the current study are available from the corresponding author upon reasonable request.
